# Work-Related Musculoskeletal Problems: A Look at How Employers View Causes

**DOI:** 10.3390/ijerph21081098

**Published:** 2024-08-20

**Authors:** Roger C. Jensen

**Affiliations:** Department of Safety, Health, and Industrial Hygiene, Lance College of Mines and Engineering, Montana Technological University, Butte, MT 59701, USA; rjensen@mtech.edu

**Keywords:** musculoskeletal, work-related, occupational injuries, occupational illnesses, repetitive motion, overexertion

## Abstract

Causation concepts for work-related musculoskeletal disorders vary among authors and academic disciplines. The major causation concepts are single-event and repetitive motion. The aim of this short communication is to share findings from a recent survey of United States employers conducted by the U.S. Bureau of Labor Statistics about work-related musculoskeletal cases, and, more specifically, about whether the employers regard the causes of their employees’ musculoskeletal problems as being from a single exposure or from multiple exposures. Recommendations are offered for using terminology consistent with employer understandings.

## 1. Introduction

This article contributes to concepts of causation and terminology for work-related musculoskeletal problems based on findings from a survey of United States employers regarding the musculoskeletal problems experienced recently by their employees.

### 1.1. Background

The field of occupational safety and health (OSH) encompasses a multitude of workplace hazards and several professional specialties, including industrial hygiene, safety/injury prevention, occupational medicine, occupational health nursing, and ergonomics. All of these professions support the goal of prevention as the most desirable approach for serving the safety and health needs of employees and minimizing the financial losses attributed to injuries and illnesses [1,2,3]. However, the professionals in these fields are not always in agreement on prevention strategies and terminology used in reference to musculoskeletal problems. For many employers, musculoskeletal problems are among the most frequent and costly types of reportable cases and workers’ compensation claims. If the safety and health manager of an enterprise recognizes the need to strengthen programs addressing musculoskeletal problems, the employer’s concepts of causation may drive the direction of efforts to curb the impact of these problems. If, for example, the employer’s concept of the problem is that musculoskeletal problems result from single exertions, then the employers may focus on redesigning the most physically demanding tasks and training employees to perform heavy tasks using biomechanically appropriate techniques. If, on the other hand, the employer’s concept is that musculoskeletal problems stem from long-term, repetitive motions, the employer may focus efforts on identifying repetitive tasks with known risk factors and redesigning those tasks to minimize known risk factors.

In a more macro view of musculoskeletal problems, different concepts of causation may affect how national organizations approach research and prevention; consider, as a hypothetical example, the U.S. National Institute for Occupational Safety and Health. Which of the numerous subordinate divisions should have the lead on musculoskeletal research? One option might be to assign musculoskeletal research to a division having staff expertise in safety/injury prevention, based on the concept that musculoskeletal problems arise from single events; another option might be to assign musculoskeletal research to a division having staff expertise in ergonomics/human factors engineering, based on the concept that musculoskeletal problems arise from repetitive motions. A decision maker might benefit from having some relevant data about how employers view the causes of musculoskeletal problems.

### 1.2. Controversy

Authors of articles about work-related musculoskeletal problems recognize two views of causation [4,5,6,7]. Regarding the single-event view, the concept is that musculoskeletal damage comes from a single, intense exertion that exceeds the physical capability of the person’s body part. Publications reflecting this perspective use the term musculoskeletal injuries [8,9,10]. The contrasting view is that a person performing a physical task repeatedly may gradually wear down their joints, spine, ligaments, tendons, or other anatomical elements until, eventually, that body part can no longer perform the task without pain [4].

Information on how these concepts are understood may be gleaned from the results of a survey of U.S. employers conducted by the U.S. Bureau of Labor Statistics (BLS) [11,12,13]. The employer respondents were asked to describe each injury, illness, or fatality their employees experienced during the recent two years according to descriptive categories of interest [14,15]. Among the descriptor attributes were the event and exposure (E&E) directly preceding the injury, illness, disorder, or fatality [13,14]. The survey items regarding overexertion cases asked if the case’s injury or illness resulted from a single episode or from multiple episodes. Responses may shed light on how employers view the source of the musculoskeletal problems incurred by employees and what terms to use when referring to musculoskeletal problems.

### 1.3. Aim

The aim of this short communication is to share findings from an examination of the recent BLS survey about occupational musculoskeletal cases, and, more specifically, to explore whether employers regard musculoskeletal problems as being the result of a single exposure or from multiple exposures.

## 2. Methods

### 2.1. Materials

BLS conducts a biennial survey of employers and makes results available through their website (BLS.gov). BLS personnel use a sampling strategy to select employers to represent the larger population of private employers in the United States. The survey asks for information on work-related injury and illness cases during the 2-year (2021–2022) period of the BLS study. The BLS survey collects various factual attributes relevant to the E&E category—not including less-than-factual psychosocial aspects, workplace climate, or managerial influence. In addition to the case information, employers must also provide employment data needed to compute incidence rates [11,12,13].

The cases used for this paper were nonfatal occupational injury and illness cases involving days away from work, restricted activity, or job transfer, commonly referred to as DART cases, which comes from the words days away, restricted, and transfer. Survey results for the years 2021–2022 are shared with the public on BLS websites in spreadsheets, graphics, and commentary [12].

Survey respondents were asked to report each case using codes in a hierarchical structure, using four placeholders for numeric codes [7,11,13,14,15]. The top level of the hierarchical structure belongs in the left-most placeholder. Table 1 lists the seven divisions having individual hierarchical structures. The number 7 in this placeholder indicates the E&E Division “overexertion and bodily reaction”. The E&E codes contain the most useful information related to causation and prevention because they inform about situations immediately preceding the harmful event [13,14].

Table 1 lists the Level I and applicable Level II categories and code numbers. For E&E code 7, the Level II column lists subcategories relevant to this project while not listing subcategories considered irrelevant, specifically, these three codes: 74, bodily conditions not elsewhere classified; 78, multiple types of overexertion and bodily reactions; and 79, overexertion and bodily reactions and exertions not elsewhere classified.

### 2.2. Analyses

The initial analysis of the E&E category 7 cases involved identifying and determining the portion of cases in each Level II subcategory. The published dataset consisted of a number of cases which the BLS statisticians projected for the entire U.S. from the survey sample. Further analyses focused on third and fourth level questions relevant to the study aim. Per a previous recommendation [10,14], a graphical form of presentation was created to concisely convey the findings in an easily appreciated manner.

## 3. Results

### 3.1. Level II Proportions

**Figure 1:**
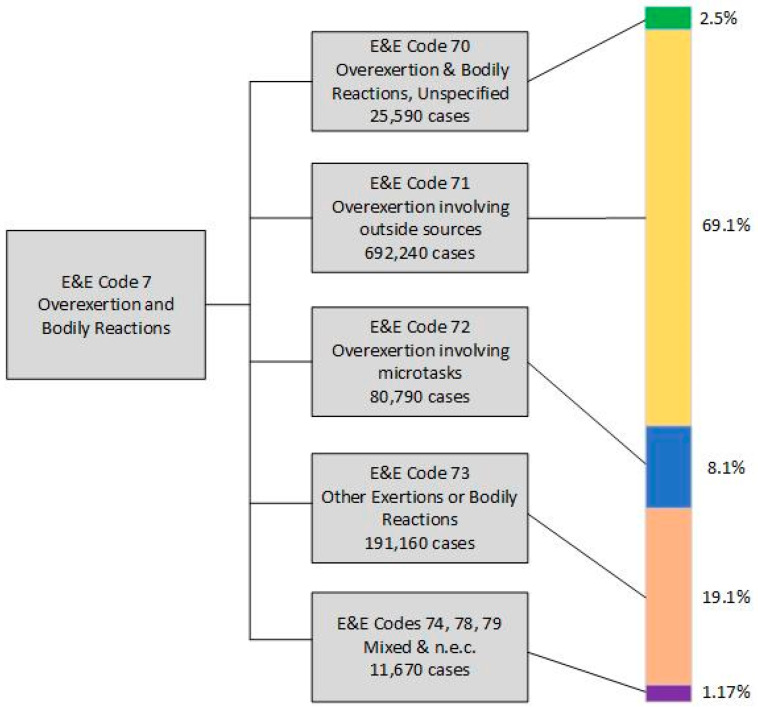
Graphic summary of cases in the BLS subcategories of E&E Division 7. The vertical bar graphic shows the portion of total cases contributed by each of the subcategories. Lines connect colors to applicable E&E category.

The graphic depiction in Figure 1 shows essential findings in the BLS E&E Division 7, grouped into subcategories 70, 71, 72, 73, 74, 78, and 79. Subcategories 74, 78, and 79 are depicted together because their information content provides no value to this project individually, and their combined contributions constituted only 1.17%. of the total.

The official title of subcategory 71, “Overexertion involving outside source”, is essentially what the industrial engineering and physical ergonomics communities call manual materials handling [4,5]. Cases in category 71 compose 69 percent of the E&E code Division 7 cases in the national data set. This broad category has third-level subcategories for lifting, lowering, pushing, pulling, turning, throwing, catching, and carrying. The largest subcategory, lifting, offers a fourth level for indicating if the injury resulted from a single episode or from multiple episodes of lifting. Respondents marked the single episode choice over 16 times more frequently than the multiple episode choice (482,510 versus 29,680 cases). Subcategory 73 contributed the second largest portion (19%) of all E&E code Division 7 cases. The cases in E&E 73 includes bending, crawling, reaching, and twisting. Within this conglomerate subcategory, respondents marked the single episode choice over 12 times more frequently than the multiple or repetitive choice (164,250 versus 13,579).

**Figure 2:**
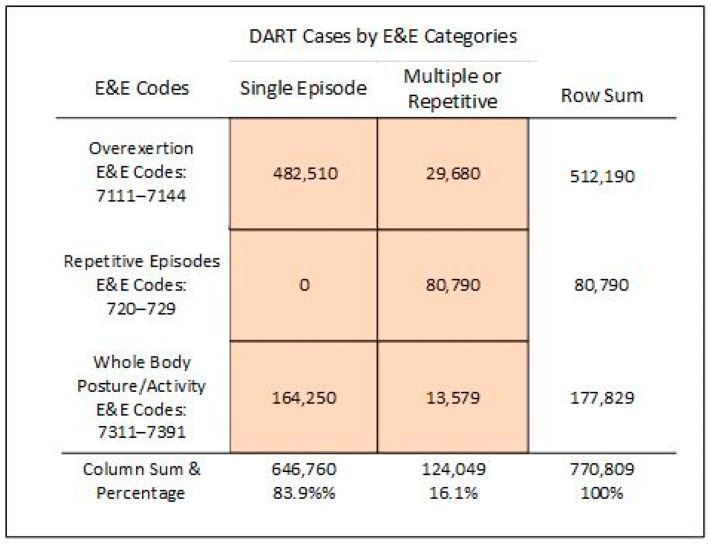
The BLS survey of employers regarding injuries and illnesses that occurred in 2021 and 2022, coded by three E&E subcategories, further split by occurring from a single episode or from multiple episodes.

To address the controversy about how to describe causes of occupational musculoskeletal cases, the BLS reports were examined to learn how the employers considered the causes of musculoskeletal problems experienced by their employees. Responses in the three rows in Figure 2 differed among the E&E categories 71, 72, and 73 in their respective proportions of cases regarded as being caused by a single event; specifically, a single episode caused 94% of overexertion cases, 0% of repetitive episode cases, and 92% of whole-body posture or activity cases.

### 3.2. Validity of Findings

Some findings in the published results strengthen the confidence that the employer’s designees for responding to the BLS survey results were able to appreciate the items about single versus multiple/repetitive causes. Referring to findings in Figure 2, causes differed among the three rows, which distinguished Level II codes 71, 72, and 73, respectively. Data in the top and lowest rows of Figure 2 involving overexertion responses show that responders were able to distinguish the two causes of musculoskeletal problems. The second finding supporting internal validity appears in the middle section of Figure 2, where all items were specifically about multiple-episode causes. These results indicate that 100% of respondents chose that, as expected, the cause was multiple episodes.

A finding supporting external validity comes from comparing the disorders identified in the survey to the affected body parts expected from the literature. Items in the survey had categories for the “nature of injury or illness”, somewhat like a layman’s diagnosis [15]. In another part of the survey, responding individuals indicated the employee’s part of body affected. An indicator of external validity would be a match between the nature of harm and body part affected. For cases coded as sprains, the expected body parts were joints held together with ligaments. The survey results were consistent with this expectation—the most common body parts identified were ankles (33%), knees (19%), back (13%), and wrist (11%). For cases coded as strains, the expectation was for body parts where muscles are heavily involved in significant exertions—back (51%) and shoulder (19%). Also consistent with the established literature [10], in cases coded in one of the soft tissue categories, the main body parts identified were as expected:

- Bursitis: knee (43%), arm (33%), and shoulder (24%).
- Stenosing tenosynovitis: hand (66%) and wrist (39%).
- Other tenosynovitis: hand (39%) and wrist (38%).
- Epicondylitis: arm (97%).
- Other non-specified tendonitis: wrist (28%) and arm (25%).
- Ganglion or cystic tumor: wrist (64%) and hand (29%).

## 4. Discussion

Survey research has both strengths and limitations. A strength of surveys in general is learning about people’s attitudes, feelings, and opinions. Most of the BLS survey items seek factual records about the employer’s injury and illness logs rather than subjective items and experiences.

The paired choice items are somewhat different in that these items ask about the source of overexertion musculoskeletal cases being multiple episodes or a single episode. Answering involves using a mix of objective facts and subjective judgment by the survey responder. The objective aspects are extracting case records from the employer’s injury and illness log, while the subjective aspects come from judging the cause without direct information in the injury and illness log. To keep this project objective, the entries by the individual responsible for recording each case in the employer’s ongoing injury/illness log were accepted as entered. That individual may have some room to describe the nature of a case as, for example, a ligament sprain or a muscle strain. For purposes of the analyses reported here, the coding category reported by the employer’s representative is accepted as representing the employers view of causation.

## 5. Conclusions

The BLS survey data provided a dataset suitable for achieving the aim of this project; specifically, this project aimed to share findings from an examination of the recent BLS survey about occupational musculoskeletal cases, and more specifically, about whether cases arose from a single episode or from multiple episodes. Based on findings from this project, the author offers four conclusions.

Conclusion 1. According to these data, the survey items on manual materials handling cases (E&E codes 7111–7114) received responses indicating approximately 6% (29,680 cases) were from multiple episodes while 94% (482,510 cases) were from single episodes.

Conclusion 2. The BLS survey included some items relevant to concepts of causation for occupational musculoskeletal problems; these were the items asking if the source of a particular musculoskeletal problem was from a single episode or from multiple episodes. If source is considered a risk factor, then the analyses described here support a finding of source as a categorical risk factor for several kinds of work-related musculoskeletal disorders. This broad conclusion is consistent with past ergonomics literature [4,5,6,7]. On a more specific level, this conclusion involves two parts: one is that repetitive work is a risk factor for work-related musculoskeletal disorders (WRMSDs), and the second is that manual material handling is a risk factor for musculoskeletal injuries as defined by OSHA (see footnote to Figure 1). Both of these conclusions provide additional support for the current thinking within the ergonomics community. Any attempt to learn more about risk factors for work-related musculoskeletal disorders from the BLS survey data is limited because it lacks comparison data on workers who did not experience a musculoskeletal injury or illness.

Conclusion 3. Data presented in this article indicate that employers recognize that injuries from manual materials handling may come from single events (94%) or from repetitive stressors (6%). Employer responses to the BLS survey suggest the term injury is appropriate for the 94 percent of musculoskeletal harms resulting from a single incident, while the term injury is inappropriate for musculoskeletal harms resulting from repetitive stresses. For the latter, appropriate terms include disorder, syndrome, and specific medical diagnostic names.

Conclusion 4. The BLS survey data are not suitable for learning about the combined involvement of long-term wear-and-tear reducing the physical capability of body parts to a point where a common physical load exceeds the body part’s tolerance, causing pain or other symptoms.

### Recommendations

Based on the BLS survey findings, the author makes two recommendations for future references to occupational musculoskeletal problems. The first is avoid using overly broad labels, such as saying that all work-related musculoskeletal problems are repetitive motion disorders or that all musculoskeletal problems are caused by single, excessive biomechanical forces on a joint or related anatomical structure. The second recommendation is to use terms precisely to match the sort of hazard associated with the work; specifically, for assembly line work and other highly repetitive work, use either repetitive motion or repetitive stress to describe the hazard of long-term repeatedly stressing the same body parts, and for general, whole-body manual work, use the term injury when referring to overexertion associated with whole body activities if the harm resulted from a single exertion or motion.

## Figures and Tables

**Table 1:** Coding levels I and II of the BLS coding system.

E&E #	Level I Division †	Level II Subcategory
1	Violence and other injuries by persons or animals	11. Intentional injury by person
		12. Injury by person—unintentional or intent unknown
		13. Animal and insect-related incidents
2	Transportation incidents	21. Aircraft incidents
		22. Rail incident
		23. Animal and other non-motorized vehicle transportation incidents
		24. Pedestrian vehicular incidents
		25. Water vehicle
		26. Roadway incidents involving motorized land vehicle
3	Fires and explosions	31. Fires
		32. Explosions
4	Falls, slips, trips	42. Falls to same level
		43. Falls to lower level
		44. Jumps to lower level
		45. Fall or jump curtailed by personal arrest system
5	Exposure to harmful substances and environments	51. Exposure to electricity
		53. Exposure to temperature extremes
		55. Exposure to other harmful substances
		56. Exposure to oxygen deficiency (n.e.c.)
6	Contact with objects and equipment	62. Struck by object or equipment
		63. Struck against object or equipment
		64. Caught in or compressed by equipment or objects
		65. Struck, caught, or crushed in collapsing structure, equipment, or material
7	Overexertion and bodily reaction	71. Overexertion involving outside forces
		72. Overexertion involving microtasks
		73. Other exertions or bodily reactions

† The U.S. Occupational Safety and Health Administration’s definition of work-related injuries, illnesses, and fatalities are those in which an event or exposure in the work environment either caused or contributed to the condition. Injuries include cases such as, but not limited to, a cut, fracture, sprain, or amputation; illnesses include both acute and chronic illnesses, such as, but not limited to, a skin disease (i.e., contact dermatitis), respiratory disorder (i.e., occupational asthma, pneumoconiosis), or poisoning (i.e., lead poisoning, solvent intoxication).

## Data Availability

The data presented in this study are available on request from the corresponding author.
